# Topics of Public Interest

**Published:** 1917-05

**Authors:** 


### TOPICS OF PUBLIC INTEREST

**The Medical Brotherhood** has temporarily suspended its activities on account of disturbed political conditions. It should be distinctly understood that this organization was in no sense political, but it has been considered wise to avoid even the active attempt to preserve a proper harmony among medical men, without regard to nationality, at a time when the purpose might be misunderstood.

**Increase in Price of Drugs.** Since the outbreak of the European War, there has been an average increase to about 5 times the original prices. Some notable increases are: resorcin 38 times; benzoic acid 31; potassium permanganate 27; digitalis 35; arnica 14; guaiacol carbonate 19; sodium benzoate 18; acetphenitidin 17; saccharin 15. In most cases the increase has not been due directly to interference with importation of drugs that cannot be raised in this country, nor even to obvious increase of demand for military purposes but solely to the fact that we have not had the gumption to collect crude drugs easily obtainable and to extract their active principles or to manufacture synthetics.

**State Board Examinations.** (Condensed from J. A. M. A. April 14). There are 89 medical colleges in the U. S. that conferred the degree of M. D. in 1916, and 50 state boards including that of the D. C. and Alaska. 60 of the colleges are recognized by all boards, 9 by all but 1-3. Class A schools yielded 75% of candidates. Class B 16%. The average failures of Class A schools was 8.5%, of Class B 24%, of Class C 29.4%. Of the 3,518 graduates of 1916, only 2,799 (79.7%) took state board examinations, but 1,316 members of classes of 1912-16 were also examined, and 315 of 1911 and previous years, making a total of 4,430 examined. Many, of course, were reexaminations, but the total has fallen off considerably on account of reciprocal recognition of license.

---

**Early Graduation of Medical Classes.** It is probable that, with the approval of the War Dept., the University of Buffalo and other medical colleges will make an effort to graduate medical classes some weeks ahead of schedule, and possible that summer courses will be instituted so as still further to anticipate the graduation date of the classes of 1918.

---

**Recent Graduates Available.** Class of 1912, 4,482 graduated, 4,345 living according to general life table; 1913, 3,981 graduated, 3,890 living; 1914, 3,594 graduated, 3,530 living; 1915, 3,536 graduated, 3,495 living; 1916, 3,518 graduated, 3,500 living. 1917, estimated, 3,367 senior students, 3,200 to be graduated. Total living 22,051. Required for military service, on basis of 7 medical men per 1,000 troops,, 1½ million beyond present army, navy and national guard, 10,500.

---

**The Charge of Grand Larceny** made by a patient against Dr. A. W. Bender of Buffalo, has been dismissed by agreement.

---

**Centenarians.** David Mosher of E. Aurora celebrated his 100th birth-day, Mch. 30; Abraham Cole the oldest resident of Ulster Co., N. Y., died at Zena, Mch. 31, aged 104; Abdour Hopshe, said to have been born in 1802 in Tripoli, died in Newburg, N. Y., Mch. 14; Mrs. Missouri Kibbe Hawkins, the widow of a Jamestown, N. Y., druggist, died in N. Y. City, April 13, aged 102.

---

**Medical Examiner.** Senator Gichrist and Assemblyman Duff have introduced a bill in the N. Y. Legislature to make this new office elective instead of based on civil service examination.

**Health of German Army.** The general morbidity rate, based on monthly reports, has been reduced from 12% for the first year of the war to 10%. Typhus has declined from 5.6% to 1.4%; diarrhoea from 2.8 to 1.8; Asiatic cholera from 0.32 to 0.24; scarlet fever from 0.18 to 0.15; tuberculosis from 2.9 to 1.7; nervous conditions from 24.3 to 21.5%. Spotted fever (probably what we call typhus, while typhus mentioned above is typhoid), intermittent (malarial?) fever and diphtheria, 0.44 together, have increased to an aggregate of 1.35. (Note that the basis of percentage of total morbidity and of the individual diseases is different). 70% of wounded are returned to active duty, 6.4 are permanently unfit for service, the remainder are used for garrison and camp duties. Of wounded sent to base hospitals, 90% are rendered fit for service, either in the fighting line or otherwise, 1% die and 9% are mustered out of service. A total of 1250 blinded have occurred since the beginning of the war. Small pox is reported to have developed from infection brought from the eastern front, 135 cases with 11 deaths having recently occurred in Berlin.

---

**Restricting Boy Labor.** A bill has been introduced into the N. Y. Legislature, prohibiting boys under 18 from blacking boots, selling papers, or working in any public place. Another bill raises the age of compulsory school attendance to 18. The legislative committee of the State Teachers' Assn. and the Lockport Board of Education oppose both. It is high time that we realized the tremendous economic disadvantages of carrying our proper reverence for education to an extreme.

---

**State Hospital Maintenance and Development.** The Sage bill has been passed by the N. Y. legislature, establishing a comprehensive plan of management and appropriating $100,000 for new buildings at Middletown, and reappropriating $300,000 from the Mohansic Hospital fund for new buildings on the Marcy site near Syracuse.

---

**Farm Lands for Perrysburg (J. N. Adam Memorial) Hospital.** Dr. Clarence L. Hyde has been authorized to rent 200 acres at $900 a year.

---

**Losses Among British Physicians.** 400 were killed and wounded in the battle of the Somme and the aggregate losses have made a shortage of medical men. The Surgeon General, U. S. Army, considers that the U. S. is the only country at present having an adequate supply of medical men for civil and military needs.

**Fire at Perrysburg Hospital,** on the night of April 10, destroyed a bull valued at $300, 7 cows and 4 horses, a carload of grain and a gas engine. The losses are supposed to be fully covered.

---

**Smoke Nuisance.** Niagara Falls has arranged through the City Manager (representing the European system and taking the place of a mayor or commission) to co-operate with the Commercial Assn. to reduce smoke to a minimum.

---

**Niagara County Tuberculosis Hospital.** With the concurrence of Dr. Otto E. Eichel, representing the State Dept. of Health, the present county hospital will be moved to the new site and will be used temporarily, pending the erection of a permanent establishment to cost $100,000.

---

**Lectures on Military Hygiene.** Major James I. Mabee, M. D., U. S. A., will give a course at the University of Buffalo, beginning April 19. This course will be open to the medical profession.

---

**Blindness in the U. S.** Director Sam. L. Rogers of the Bureau of the Census, reports that 57,272 blind were enumerated (for registration area?). Detailed statistics have been compiled from replies from 29,242 cases, having an average duration of 16 years. 6.6% were born blind, 11.6% (including congenital cases) had been blind since the first year of life, 16.4% had been blind since the first five years, 30.8% were blind by the end of the 20th year. 47.4% became blind between the ages of 20 and 64 and 21.8% after this age. 51.4% of males and 41.8% of females became blind between the ages of 20 and 64, the difference being due to occupational causes. Blindness is a bar to marriage and, obviously more so for females, since sentimental reasons outweigh economic. About 1/3 of the males who became blind between the 15th and 19th years were married and about 1/5 of the females.

---

**Medico-Military Materiel.** The Military Surgeon asks us to co-operate in calling attention to the fact that it will take much longer and cost more to accumulate materiel than men, that, for example, it will require nearly a year to obtain the materiel for an army of 750,000 men and that this will cost $10 per man. This fact has been attested by experience in previous wars. (However, it should be borne in mind that the factories for armament and ammunition have never been so well equipped as at present and that, with the development of manufacture in Europe countries to care for their own needs, this source is almost immediately available for our own country. We are also exceedingly well supplied with accessory military materiel, such as automobiles, are almost independent of foreign supplies of raw material (which becomes materiel by manufacture) and that even such raw material as sugar and rubber which we cannot raise sufficiently or at all, is more easy of import into this country than most others. A somewhat amusing story is told that illustrates that the fear of great delay in securing materiel may be exaggerated. An order was placed for 35,000 shells and, accompanying the order, was a letter asking the firm to secure a boarding place for the inspector while the order was being filled. The firm replied that he could wait almost anywhere as their average daily output was 40,000).

---

**Military Preparedness of the N. Y. State Dept. of Health.** All state health officers, numbering about 1000, will be placed at the service of the military authorities for making preliminary medical examinations of recruits. The Division of Laboratories and Research is assembling a mobile laboratory unit for diagnostic service. A large stock of bacterial vaccines, etc., is being prepared. 10,000 doses of vaccines against typhoid, paratyphoid and dysentery have already been supplied for state troops. Small pox vaccine for 3,000 has been issued. Nearly 5,000 c.c. of anti-pneumonia serum was used in over 400 cases on the Texas border. An outfit is being prepared to furnish calcium hypochlorite in convenient form to disinfect 50 gallons of water at once and to remove the taste by fixing the excess of chlorin.

---

**The U. S. Civil Service Commission** announces an examination, open to men and women, for medical interne. A vacancy exists at St. Elizabeth's Hospital, Washington, at $900 and maintenance. Other vacancies will be filled from the eligible list. Applicants must not have graduated earlier than 1915, unless they have been continuously engaged in hospital, laboratory or research work in neurology and psychiatry. Examinations will be held on June 6 at various cities. (Note: As similar announcements are often received too late for publication before the examination, physicians desiring to enter the government service in any capacity, are advised to file their names with the editor).

---

**Children in War Time.** The Children's Bureau of the U. S. Dept. of Labor, calls attention to the increase in juvenile delinquency in foreign countries under the excitement of war and with mothers called from domestic to industrial duties, to the effect of overwork and deprivation of the mother upon the mortality and morbidity of infants, even antedating birth, and is planning to avoid such dangers for our own country. The particular need of inforcing laws regarding child labor and school attendance and of safe-guarding children from both physical and moral dangers, is emphasized. It is stated that more women between the ages of 15 and 45 die from conditions directly or indirectly but plainly connected with child birth, than from any other cause except tuberculosis (which also is often due to the strain of pregnancy). It is estimated that 15,000 maternal deaths and 75,000 deaths of infants occur annually, from preventable causes, the latter depending largely on the death or disablement of the mothers.

---

**Rockefeller Foundation Appropriations for 1916** amounted to over $8,000,000 of which a little over 2½ millions were for war relief. We recommend to this organization, as a very practical philanthropy, the benefits of whose application would be widely distributed, an appropriation to rebate 10 cents a gallon on gasoline, which would bring the net cost of this product about to the high normal price. There are more than 3½ automobiles in the country, averaging something like 1000 gallons a piece on an estimate of 10,000 miles travel at an average of 10 miles to the gallon. This estimate is probably high but compensated for by motor boats, stationary engines and the use of gasoline for cleaning. However, the excess price comes to something like 350 millions. It is a pertinent though somewhat ungracious question to ask whether the 8 millions to philanthropy is, in any sense, part of this 350 millions.

---

**Anthrax.** The U. S. Dept. of Agriculture states that some centuries ago, this disease caused the death of 60,000 human beings in Europe. Its present small human death rate is believed to be due to a diminution of virulence. The modern treatment consists in the injection of 10 c.c. of anti-anthrax serum beneath the skin of one side of a large animal and 1 c.c. of spore vaccine on the opposite side. For sheep, which are peculiarly susceptible, the dosage is 1/4 these amounts. Anthrax may be borne by flies, a single fly bite being sufficient to infect a horse. Deep burial with avoidance of direct drainage into streams is efficient by imposing anaerobic conditions. If carcasses have lain on the surface, they should be burned, the fire being of sufficient magnitude to disinfect the soil.

